# Treatment type may influence degree of post-dislocation shoulder osteoarthritis: a systematic review and meta-analysis

**DOI:** 10.1007/s00167-020-06263-3

**Published:** 2020-09-16

**Authors:** Lukas P. E. Verweij, Erik C. Pruijssen, Gino M. M. J. Kerkhoffs, Leendert Blankevoort, Inger N. Sierevelt, Derek F. P. van Deurzen, Michel P. J. van den Bekerom

**Affiliations:** 1 Department of Orthopedic Surgery, Amsterdam Movement Sciences, Amsterdam UMC, location AMC, University of Amsterdam, Meibergdreef 9, 1105 AZ Amsterdam, The Netherlands; 2 Academic Center for Evidence-based Sports Medicine (ACES), Amsterdam UMC, Amsterdam, The Netherlands; 3 Amsterdam Collaboration for Health and Safety in Sports (ACHSS), International Olympic Committee (IOC) Research Center, Amsterdam UMC, Amsterdam, The Netherlands; 4 Department of Orthopedic Surgery, Shoulder and Elbow Unit, Onze Lieve Vrouwe Gasthuis, Amsterdam, The Netherlands; 5 Specialized Center of Orthopedic Research and Education (SCORE), Xpert Orthopedics, Amsterdam, The Netherlands

**Keywords:** Traumatic, Anterior shoulder instability, Instability, Osteoarthritis, Non-operative, Stabilization surgery

## Abstract

**Purpose:**

Age at primary dislocation, recurrence, and glenoid bone loss are associated with development of osteoarthritis (OA). However, an overview of OA following traumatic anterior shoulder instability is lacking and it is unclear to what degree type of surgery is associated with development of OA in comparison to non-operative treatment. The aim of this study was to determine the degree of OA at long-term follow-up after non-operative and operative treatments for patients with anterior shoulder instability. Surgery is indicated when patients experience recurrence and this is associated with OA; therefore, it was hypothesized that shoulders show a higher proportion or degree of OA following operative treatment compared to non-operative treatment.

**Methods:**

A literature search was performed in the PubMed/Medline, EMBASE, and Cochrane databases. Articles reporting the degree of OA that was assessed with the Samilson–Prieto or Buscayret OA classification method after non-operative and operative treatment for anterior shoulder instability with a minimum of 5 years follow-up were included.

**Results:**

Thirty-six articles met the eligibility criteria of which 1 reported the degree of OA for non-operative treatment and 35 reported the degree of OA for 9 different operative procedures. A total of 1832 patients (1854 shoulders) were included. OA proportions of non-operative and operative treatments are similar at any point of follow-up. The Latarjet procedure showed a lower degree of OA compared to non-operative treatment and the other operative procedures, except for the Bristow procedure and Rockwood capsular shift. The meta-analyses showed comparable development of OA over time among the treatment options. An increase in OA proportion was observed when comparing the injured to the contralateral shoulder. However, a difference between the operative subgroups was observed in neither analysis.

**Conclusion:**

Non-operative and operative treatments show similar OA proportions at any point of follow-up. The hypothesis that shoulders showed a higher proportion or degree of OA following operative treatment compared to non-operative treatment is not supported by the data. Operative treatment according to the Latarjet procedure results in a lower degree of OA compared to other treatments, including non-operative treatment.

**Level of evidence:**

IV.

**Electronic supplementary material:**

The online version of this article (10.1007/s00167-020-06263-3) contains supplementary material, which is available to authorized users.

## Introduction

General risk factors for development of osteoarthritis (OA) include genetic predisposition, female gender, old age, and trauma [5, 32, 49, 50, 60]. OA of the shoulder can be painful, and lead to a decrease in range of motion, limiting shoulder function, which may lead to shoulder arthroplasty as an intervention to treat the pain and reduce the limitations [3, 52]. Studies have shown a higher degree of OA following anterior shoulder dislocation and stabilization surgery compared to the contralateral healthy shoulder [9, 30, 54]. A shoulder dislocation is a commonly established diagnosis at the emergency department, with a reported incidence of 23.9 per 100,000 person-years [30, 67]. The incidence of an anterior shoulder dislocation decreases with age, is higher in males than in females, and is higher when participating in contact and overhead sports in comparison to other types of sports [16]. When the patient experiences (recurrent) instability that affects activities of daily living, work, or sports participation, an operative stabilization procedure is indicated. Several stabilization procedures have been proposed, ranging from soft-tissue procedures to bony augmentation procedures [6]. A soft-tissue procedure, such as the Bankart repair, is usually indicated when little glenoid bone loss is present [15]. A bony augmentation procedure, for example a Latarjet procedure or an Iliac crest bone graft procedure, is usually indicated in case of extensive damage to the glenohumeral joint or excessive glenoid bone loss [51]. Both soft-tissue and bone augmentation procedures can be performed arthroscopically or with an open procedure. Plath et al. showed that an arthroscopic Bankart procedure resulted in similar long-term OA rates compared to an open procedure and non-operative treatment [48]. Age at primary dislocation, recurrence, and glenoid bone loss are associated with development of OA. However, it is unclear to what degree type of surgery is associated with development of OA in comparison to non-operative treatment [23, 25, 26, 28]. In addition, a patient will gain relatively more stability following a bone augmentation procedure in comparison to a soft-tissue procedure. To what degree this is associated with development of OA is unclear as well [7, 36].

To assess the severity of OA following a shoulder dislocation, multiple classification methods have been proposed. Using plain radiography, these methods generally consist of four or five grades of OA severity ranging from “no OA” to “severe OA” [11, 55, 56]. Severity is determined by joint space narrowing and the presence of osteophytes [11, 55, 56]. Long-term degrees of OA for other orthopedic pathologies, such as knee osteoarthritis following an anterior cruciate ligament defect, have been well described [38]. However, it remains unclear if the treatment type for anterior shoulder instability influences degree of OA [46]. If the degree of OA is influenced by treatment type in any way, it can assist both clinicians and patients in the decision-making process.

The aim of this study was to determine the degree of OA at long-term follow-up after non-operative and operative treatment for patients with anterior shoulder instability. Surgery is indicated when patients experience recurrence and this is associated with OA; therefore, it was hypothesized that shoulders show a higher proportion or degree of OA following operative treatment compared to non-operative treatment.

## Materials and methods

This systematic review focused on glenohumeral OA after non-operative and operative treatment for anterior shoulder instability. The guideline and algorithm of the Preferred Reporting Items for Systematic Reviews and Meta-Analyses (PRISMA) were used [40]. The review was registered in PROSPERO (https://www.crd.york.ac.uk/prospero/; registration number CRD42020141008).

### Literature search and study selection

A literature search was performed in the PubMed/Medline, EMBASE, and Cochrane databases on the 22^nd^ of July 2019. The search was performed with the assistance of a clinical librarian and was not limited to year of publication. The search terms are listed in the appendix. Articles written in the English, Dutch, German, or French language were included. Titles and abstracts were screened by two authors (LV and EP). Studies that met the inclusion criteria underwent full-text screening by the same authors. Any disagreement was resolved by discussion and consensus.

### Inclusion and exclusion criteria

Articles that reported degree of OA after non-operative or operative treatments for anterior shoulder instability with a minimum of 5 years follow-up were included. Controlled trials and prospective or retrospective cohort studies were included. Studies that used classification systems other than Samilson–Prieto or its modified version by Buscayret were excluded. Furthermore, studies that did not use plain radiograph to assess OA with these classifications were excluded. Studies that did not report original data, animal studies, and cadaveric studies were excluded. When the same patient group was used, the article with the longest follow-up was included for analysis.

### Methodological quality assessment

The quality of the studies was assessed by the methodological index for non-randomized studies (MINORS) [59]. By scoring 0 (not reported), 1 (reported but inadequate), or 2 (reported and adequate) at each item, studies could get a total score of 16 for non-comparative studies and 24 for comparative studies. Any disagreement was resolved by discussion and consensus.

### Data extraction

The extracted primary outcome measure was degree of OA according to both the Samilson-Prieto and Buscayret OA classification after non-operative or operative treatment for anterior shoulder instability. If the authors had assessed classification of OA on multiple radiographic views, a weighted average score of the different views was calculated. Furthermore, secondary outcome measures were extracted including type of surgery, gender, functional outcome (Rowe score), recurrent dislocation, age at surgery, and length of follow-up. The data were extracted by the lead author (LV). A random sample of ten articles was independently assessed by the second author (EP) and checked for accuracy.

### OA classification

**Fig. 1:**
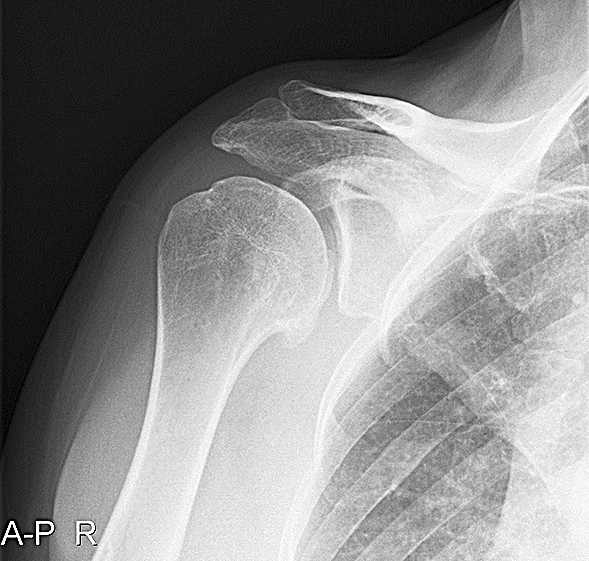
Osteoarthritis (OA) following a Bankart repair with > 10 years follow-up. This figure demonstrates an example of OA in the shoulder. The shoulder has been classified as Samilson–Prieto grade II

The Samilson–Prieto classification is a radiographical classification system which categorizes OA in 4 categories comprising (0) no OA, (I) osteophytes measuring < 3 mm in greatest distance diameter, (II) osteophytes measuring between 3 and 7 mm in greatest distance diameter and slight glenohumeral joint irregularity, and (III) osteophytes measuring > 7 mm in greatest distance diameter, narrowing of the glenohumeral joint and sclerosis (Fig. 1). For the modified version, Buscayret added one extra category by splitting the category III of the original version into the categories (III) osteophytes measuring > 7 mm in greatest distance diameter, narrowing of the glenohumeral joint and sclerosis, and (IV) complete obliteration of the glenohumeral joint with or without osteophytes. For the purpose of pooling, these two categories were merged again [11, 56]. Furthermore, if authors reported that some of their patients received arthroplasty treatment of the shoulder or that the shoulder showed complete glenoid erosion, they were considered to be grade III.

### Statistical analysis

To perform meta-analyses, data of the primary outcome were pooled. For calculation of pooled proportions and Relative Risks (RR) of OA, the Samilson–Prieto classification was dichotomized by merging all categories describing signs of OA (categories I, II, and III) as OA. Category 0 was considered as no OA. Patient characteristics and follow-up were pooled by calculation of weighted means and pooled standard deviations (SD). If the standard deviation was not reported, it was estimated with the range and the sample size according to Walter et al. [64]. Furthermore, if the mean was not reported, it was estimated using the median, range, and sample size according to Hozo et al. [31]. Comparisons of the degree of OA were performed by the use of Mann–Whitney U tests; proportions were compared by the use of Chi squared tests. Review Manager version 5.3 (the Nordic Cochrane Center, Copenhagen, Denmark) was used to calculate RR with 95% CI. Heterogeneity between studies was assessed by the use of X^2^ and I^2^ statistic. I^2^ > 50% was considered as substantial heterogeneity [24]. IBM SPSS Statistics 25 (IBM Corp., Armonk, NY) was used to perform other statistical analyses.

## Results

### Screening and study characteristics

**Fig. 2:**
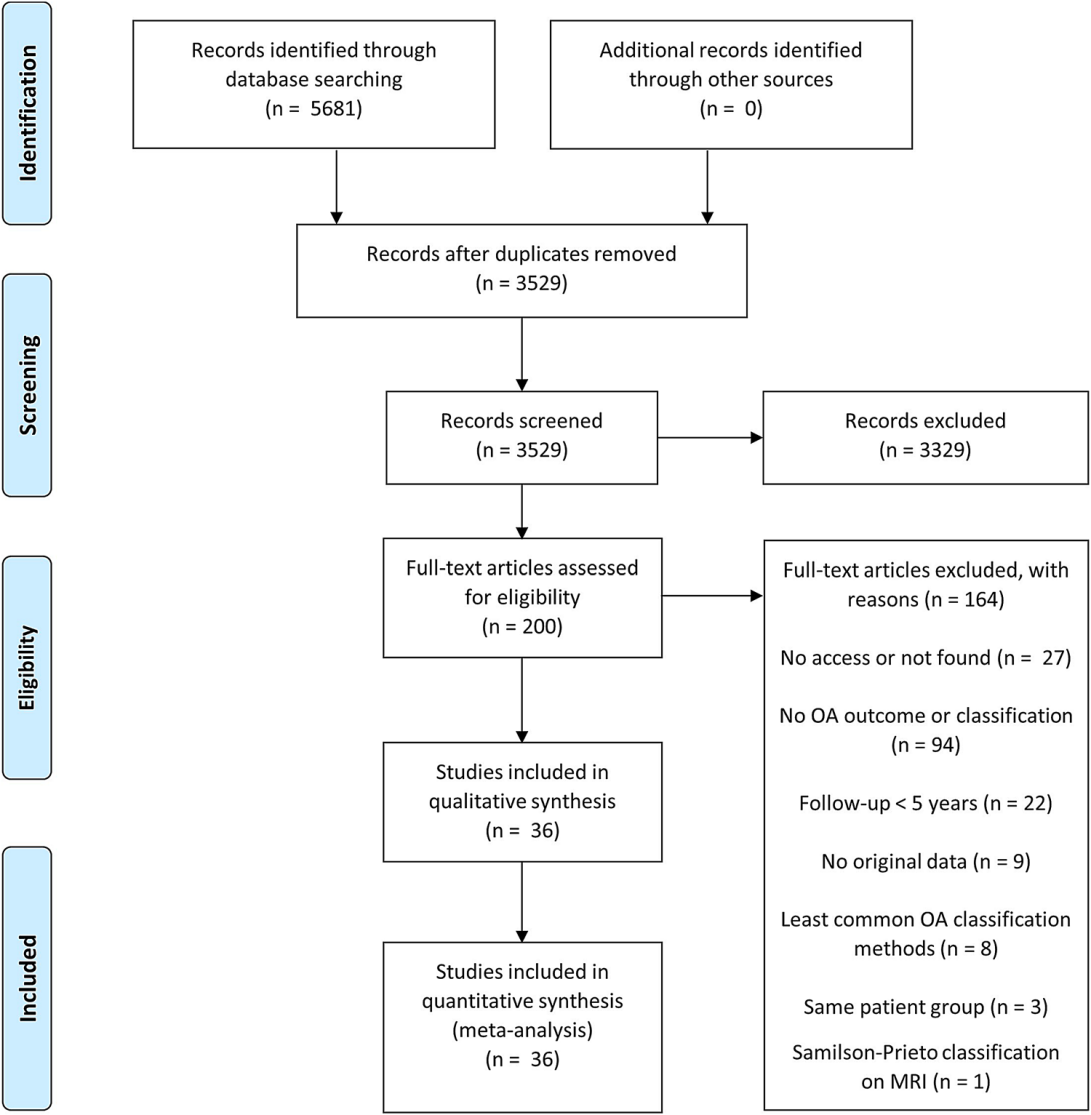
Flow diagram

The literature search resulted in 3529 articles for title and abstract screening after deduplication (Fig. 2). The full-text analysis was performed on 200 articles after title and abstract screening, resulting in 36 articles for inclusion in the analysis. Reasons for exclusion are listed in Fig. 2. In the included studies, a total of 1832 patients (1854 shoulders) were included. The sample size ranged from 9 to 161 shoulders. The follow-up ranged from 5 to 57 years. The included studies were published between 1994 and 2019. The included studies reported degree of OA for 1 type of non-operative treatment, which included 161 shoulders for conservative treatment (Tables 1, 2) [28]. Furthermore, the included studies reported degree of OA for 9 types of operative treatment, which included a total sample of 663 shoulders for the Bankart repair [4, 12, 13, 19, 20, 30, 34, 41, 44, 47, 48, 63, 69], 515 shoulders for the Latarjet procedure [8, 14, 22, 29, 30, 37, 39, 45, 58, 65], 220 shoulders for the Eden–Hybinette procedure [9, 35, 53, 54, 66], 112 shoulders for the Putti-Platt procedure [35, 62, 68], 75 shoulders for the Iliac crest bone graft procedure [42, 61], 11 shoulders for the Bristow procedure [57], 45 shoulders for the Du Toit procedure [70], 25 shoulders for Weber’s rotational osteotomy [18], and 27 shoulders for the Rockwood capsular shift (Tables 1, 2) [43]. The MINORS quality assessment ranged from 4 to 11 (median 8; IQR 8–9) of the 16 points for non-comparative studies (Table 1). The comparative studies scored 16 of the 24 points. The mean Rowe score ranged from 80.0 to 95.0 and the percentage of recurrent dislocation ranged from 0 to 41% (Table 1). Because of the small differences in quality scores between the studies, no weighing of the outcomes was applied in the analyses.

**Table 1:** Characteristics per procedure

Author	Year	Design	Sample size	% Male	Mean age at surgery	Mean FU in years	Procedure	Open/ arthroscopic	Rowe score	% Recurrent dislocation	% OA	MINORS score
Hovelius et al. [30]	2001	R	26	85	26.3 ± 5.6	17.5 ± 1.0	Bankart	Open	–	4%	62%	8/16
Pelet et al. [47]	2006	R	30	80	23.6 ± 8.1	29.0 ± 5.3	Bankart	Open	80.0 ± 16.5	10%	63%	8/16
Castagna et al. [12]	2010	R	31	87	26.3 ± 7.1	10.9 ± 1.4	Bankart	Arthroscopic	80.1 ± 17.2	13%	39%	8/16
Franceschi et al. [19]	2011	R	55	83	27.6 ± 5.4	8.0 ± 1.5	Bankart	Arthroscopic	–	8%	22%	9/16
Zaffagnini et al. [69]	2012	R	49	–	35.0 ± 8.0	13.7 ± 2.2	Bankart	Arthroscopic	85.0 ± 22.6	12%	37%	16/24
Zaffagnini et al. [69]	2012	R	33	–	38.0 ± 10.0	15.7 ± 2.2	Bankart	Open	83.2 ± 24.4	9%	45%	16/24
Kavaja et al. [34]	2012	R	74	74	29.0 ± 9.2	13.0 ± 0.8	Bankart	Arthroscopic	–	26%	68%	8/16
Gamulin et al. [20]	2014	R	33	71	27.2 ± 9.3	12.9 ± 0.9	Bankart	Open	–	14%	45%	10/16
Chapus et al. [13]	2015	P	21	95	20.5 ± 3.4	9.7 ± 1.1	Bankart	Arthroscopic	86.0 ± 22.0	25%	14%	9/16
Plath et al. [48]	2015	R	100	77	27.7 ± 7.7	13.0 ± 1.5	Bankart	Arthroscopic	–	14%	69%	8/16
Moroder et al. [41]	2015	R	26	75	26.7 ± 9.4	22.0 ± 1.2	Bankart	Open	88.7 ± 12.0	18%	50%	8/16
Neviaser et al. [44]	2017	R	91	80	31.0 ± 9.7	5.0 ± 3.8	Bankart	Open	91.4 ± 6.1	1%	47%	8/16
Berendes et al. [4]	2018	R	39	82	31.0 ± 6.7	21.0 ± 2.3	Bankart	Open	85.0 ± 17.3	10%	51%	16/24
van Gastel et al. [63]	2019	R	55	72	30.0 ± 8.0	13.1 ± 1.5	Bankart	Arthroscopic	–	10%	60%	8/16
Singer et al. [58]	1995	R	14	–	25.0 ± 5.3	20.5 ± 0.7	Latarjet	Open	–	0%	71%	5/16
Hovelius et al. [30]	2001	P	30	80	28.4 ± 7.4	15.1 ± 0.2	Latarjet	Open	–	3%	30%	8/16
Hovelius et al. [29]	2006	P	115	81	29.3 ± 8.4	15.0 ± 0.5	Latarjet	Open	89.5 ± 10.9	3%	40%	10/16
Shih et al. [65]	2012	R	28	75	23.3 ± 3.4	9.0 ± 0.8	Latarjet	Open	88.3 ± 6.1	0%	32%	7/16
Neyton et al. [45]	2012	R	37	100	23.4 ± 3.7	12.0 ± 3.2	Laterjet	Open	93.0 ± 9.2	0%	30%	6/16
Lädermann et al. [37]	2013	R	110	70	28.4 ± 8.5	16.2 ± 2.4	Latarjet	Open	–	2%	39%	9/16
Bouju et al. [8]	2014	R	70	71	26.7 ± 8.4	13.0 ± 2	Latarjet	Open	–	1%	7%	6/16
Mizuno et al. [39]	2014	R	60	79	29.4 ± 8.8	20.0 ± 0.8	Latarjet	Open	–	3%	33%	9/16
Gordins et al. [22]	2015	R	31	74	27.0 ± 4.4	33.0 ± 5.1	Latarjet	Open	–	3%	65%	8/16
De L’Escalopier et al. [14]	2018	R	20	–	26.5 ± 8.0	16.3 ± 2.4	Latarjet	Open	91.8 ± 9.9	0%	15%	8/16
Wildner et al. [66]	1994	R	56	87	30.0 ± 8.9	15.0 ± 2.8	Eden–Hybinette	Open	–	–	79%	6/16
Konig et al. [35]	1997	R	9	–	27.3 ± 4.0	26.9	Eden–Hybinette	Open	–	–	89%	4/16
Rachbauer et al. [53]	2000	R	36	79	27.0 ± 7.6	15.0 ± 3.5	Eden–Hybinette	Open	–	31%	89%	8/16
Brox et al. [9]	2003	R	45	58	25.0 ± 9.3	14.0 ± 3.1	Eden–Hybinette	Open	90.0 ± 13.2	4%	56%	9/16
Rahme et al. [54]	2003	R	74	74	26.0 ± 9.4	29.0 ± 3.1	Eden–Hybinette	Open	84.0 ± 15.0	21%	47%	8/16
Konig et al. [35]	1997	R	26	–	24.0 ± 3.0	26.9	Putti–Platt	Open	–	–	58%	4/16
Van der Zwaag et al. [62]	1999	R	66	74	26.9	22.3 ± 6.3	Putti–Platt	Open	–	–	61%	8/16
Zaffagnini et al. [68]	2000	R	20	78	24.1 ± 7.0	27.1 ± 2.7	Putti–Platt	Open	94.5 ± 9.4	0%	35%	7/16
Steffen et al. [61]	2013	R	40	83	25.0 ± 4.6	9.2 ± 3.6	Iliac crest	Open	–	3%	50%	8/16
Moroder et al. [42]	2018	R	35	88	30.0 ± 10.0	18.0 ± 2.0	Iliac crest	Open	94.0 ± 10.0	3%	74%	8/16
Schroder et al. [57]	2006	R	11	–	20.5 ± 1.3	26.4 ± 1.1	Bristow	Open	81.8 ± 29.9	–	45%	9/16
Zaffagnini et al. [70]	2007	R	45	79	24.0 ± 5.1	35.0 ± 5.8	Du Toit	Open	95.0 ± 7.8	3%	60%	10/16
Flury et al. [18]	2007	R	25	68	–	14.5 ± 2.4	Weber’s rotation osteotomy	Open	84.0 ± 17.8	6%	85%	8/16
Murena et al. [43]	2016	R	27	88	27.0 ± 11.0	13.0 ± 2.0	Rockwood capsular shift	Unclear	91.5 ± 14.7	18%	15%	6/16
Hovelius et al. [28]	2009	P	161	80	–	25.0 ± 0.0	Conservative	–	–	41%	60%	10/16

This table, sorted per procedure (*n* = 39), shows the characteristics of the long-term follow-up of the included studies (*n *= 36). Three studies reported long-term follow-up of two procedures; these studies are shown twice in this table. When the mean or standard deviation were not presented in the included articles, they were calculated according to Hozo et al. and Walter et al

*OA* osteoarthritis, *FU* follow-up, *R* retrospective, *P* prospective

**Table 2:** Characteristics of procedures

Procedure	Studies (n)	Sample size (n)	Weighted follow-up in years	Weighted age at surgery	SP Grade 0	SP Grade I	SP Grade II	SP Grade III	P- value*	Weighted Rowe score	Pooled % recurrent dislocation	Pooled % OA
Bankart [4, 12, 13, 19, 20, 30, 34, 41, 44, 47, 48, 63, 69]	13	663	14.9 ± 2.0	29.1 ± 8.0	49.0%	34.8%	10.6%	5.6%	*P* < 0.001	86.1 ± 15.4	11%	51%
Latarjet [8, 14, 22, 29, 30, 37, 39, 45, 58, 65]	10	515	16.3 ± 1.7	27.6 ± 7.5	66.0%	25.0%	5.0%	3.9%	–	90.2 ± 9.7	2%	34%
Eden-Hybinette [9, 35, 53, 54, 66]	5	220	19.7 ± 3.1	27.0 ± 8.8	34.5%	33.2%	18.6%	13.6%	*P* < 0.001	86.3 ± 14.4	18%	65%
Putti-Platt [35, 62, 68]	3	112	25.7 ± 1.9	24.2 ± 4.2	44.6%	32.1%	16.1%	7.1%	*P* < 0.001	94.5 ± 9.4	0%	55%
Iliac crest [42, 61]	2	75	13.3 ± 2.8	27.3 ± 7.1	38.7%	54.7%	5.3%	1.3%	*P* < 0.001	94.0 ± 10.0	3%	61%
Bristow [57]	1	11	26.4 ± 1.1	20.5 ± 1.3	54.5%	9.1%	0.0%	36.4%	*P* = 0.14	81.8 ± 29.9	–	45%
Du Toit [70]	1	45	35 ± 5.8	24 ± 5.1	40.0%	48.9%	8.9%	2.2%	*P* = 0.001	95.0 ± 7.8	3%	60%
Weber’s rotational osteotomy [18]	1	25	14.5 ± 2.4	–	20.0%	40.0%	24.0%	52.0%	*P* < 0.001	84.0 ± 17.8	6%	85%
Rockwood capsular shift [43]	1	27	13 ± 2	27.0 ± 11.0	85.2%	3.7%	3.7%	7.4%	*P* = 0.08	91.5 ± 14.7	18%	15%
Conservative [28]	1	161	25.0 ± 0.0	–	39.8%	33.5%	9.3%	17.4%	*P* < 0.001	–	41%	60%

The pooled data of the Samilson–Prieto (SP) grading for osteoarthritis (OA) for the treatment options

*This *p* value is calculated by comparing the SP classification outcome of the treatment option with the lowest pooled OA proportion (Latarjet) with the other treatment options with a Mann–Whitney U test

*SP* Samilson–Prieto, *OA* osteoarthritis

### Comparison of OA among non-operative and operative treatment options

**Fig. 3:**
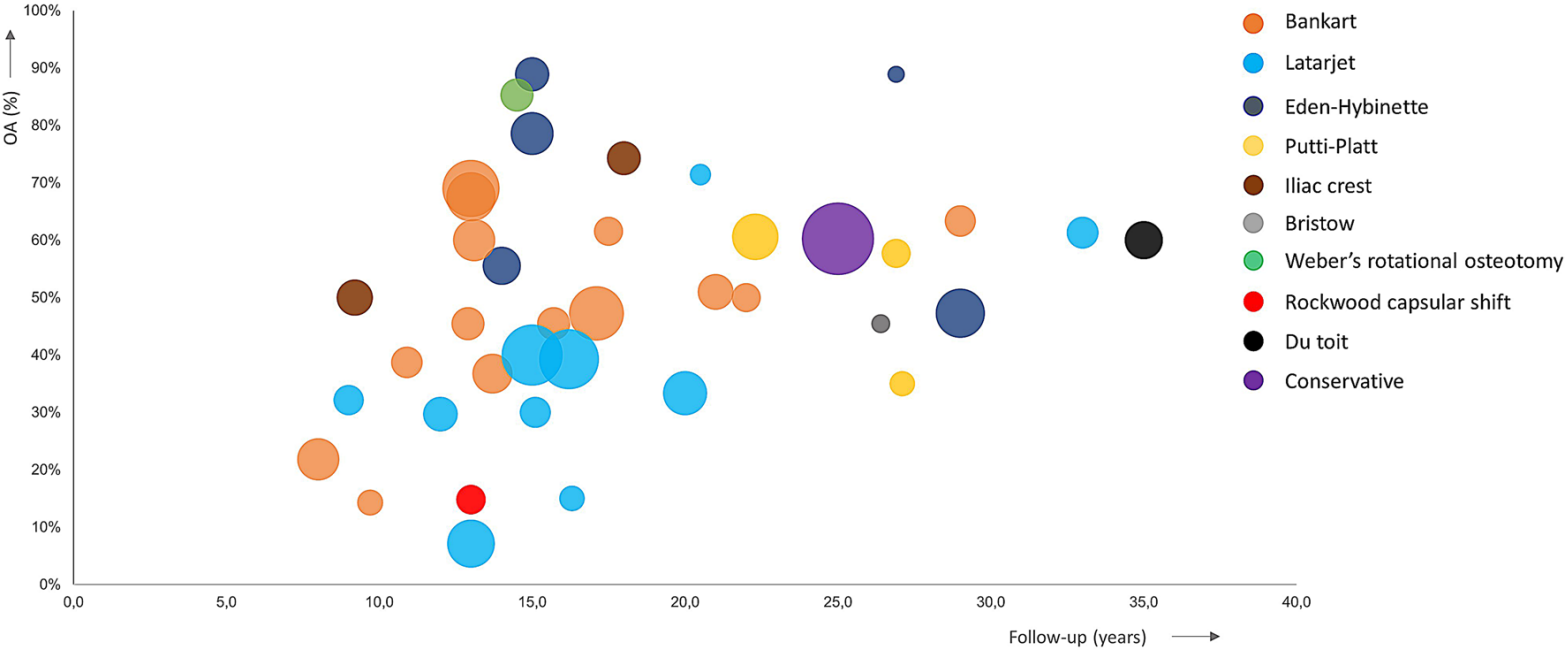
Percentage of osteoarthritis after operative or non-operative treatments. This figure shows the percentage of osteoarthritis for each individual procedure (*n* = 39) of the included studies (*n* = 36). Three studies reported long-term follow-up of two procedures; these studies are shown twice in this figure. The color of the circle matches a procedure that can be found in the legend. The size of the circle is dependent on sample size and increases in size with a larger sample size. OA proportion was calculated by merging all categories describing signs of OA (categories I, II, and III) as OA. OA = Osteoarthritis

OA proportion was calculated by merging all categories describing signs of OA (categories I, II, and III) as OA. Non-operative and operative treatments show similar OA proportions at any point of follow-up (Fig. 3). The Latarjet procedure seems to show a slightly lower proportion of OA compared to other treatment options.

Comparison of the degree of OA according to the Samilson–Prieto scale among treatment modalities revealed that the Latarjet procedure shows a significantly lower degree of OA compared to the Bankart repair (*P* < 0.001), the Eden–Hybinette procedure (*P* < 0.001), the Putti–Platt procedure (*P* < 0.001), the Iliac crest procedure (*P* < 0.001), the Du Toit procedure (P = 0.001), the Weber’s rotational osteotomy (*P* < 0.001), and conservative treatment (*P* < 0.001; Table 2). There was no statistically significant difference between the Latarjet procedure and the Bristow (n.s.) and the Latarjet procedure and the Rockwood capsular shift procedure (n.s.; Table 2). Degree of OA following arthroscopic surgery was only reported for the Bankart repair. Seven studies reported degree of OA following arthroscopic surgery, six studies following open surgery, and one study compared both surgery techniques. A significant difference in degree of OA between an open or arthroscopic Bankart repair was not observed (n.s., Table 1).

### Proportion of OA preoperatively and at follow-up

**Fig. 4:**
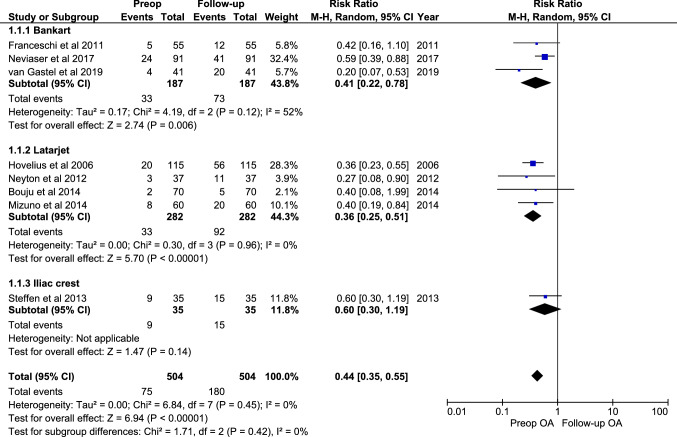
Meta-analysis of preoperative vs follow-up for proportion of osteoarthritis (OA). This meta-analysis shows the ratio of the studies (*n* = 8) that reported degree of OA preoperatively and at follow-up. The proportion of OA was calculated to perform the meta-analysis. Furthermore, the difference between the subgroups is determined. OA proportion was calculated by merging all categories describing signs of OA (categories I, II, and III)

Eight studies reported consistent data for degree of OA preoperatively and at follow-up following treatment. Overall, a higher proportion of OA post-operative compared to pre-operative was observed for the Bankart repair and the Latarjet groups, but not for the iliac crest group (Fig. 4). The likelihood of developing OA was not statistically significantly different between the treatment groups Bankart, Latarjet, and Iliac crest (n.s.; Fig. 4).

### Proportion of OA of the injured versus contralateral shoulder

**Fig. 5:**
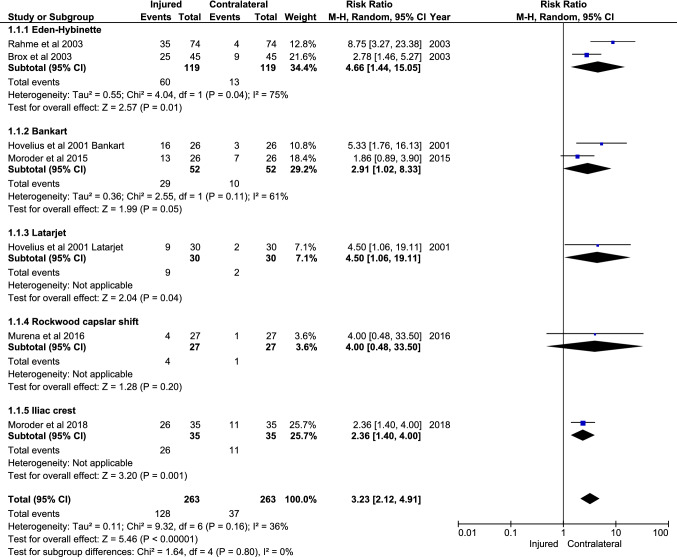
Meta-analysis of injured vs contralateral shoulder for proportion of osteoarthritis (OA). This meta-analysis shows the ratio of the studies (*n* = 7) that reported the degree of OA for the injured and contralateral shoulder for a procedure. The proportion of OA was calculated to perform the meta-analysis. Furthermore, the difference between the subgroups is determined. OA proportion was calculated by merging all categories describing signs of OA (categories I, II, and III)

Seven studies reported consistent data for degree of OA for both the injured and contralateral (healthy) shoulder. Overall, a higher proportion of OA for the injured shoulder compared to the contralateral shoulder was observed for the Bankart repair, the Latarjet, Eden–Hybinette, and iliac crest groups, but not for the Rockwood capsular shift (Fig. 5). There was no statistically significant difference in proportion of OA between the subgroups Eden–Hybinette, Bankart, Latarjet, Rockwood capsular shift, and Iliac crest (n.s.; Fig. 5).

## Discussion

The most important finding of the present study was that proportion of OA seems comparable between the various treatments and may be unrelated to treatment type with a follow-up ranging from 5 to 35 years. However, the analyses of the data revealed a statistically significant lower degree of OA after the Latarjet procedure compared to other treatment types at follow-up, except the Bristow procedure and Rockwood capsular shift. A difference in degree of OA between an open or arthroscopic Bankart repair was also not observed. In addition, the meta-analyses showed that the likelihood of developing OA after a Bankart and Latarjet was similar and an overall increase in proportion of OA was observed for the injured shoulder compared to the contralateral shoulder for the Bankart repair, the Latarjet, Eden–Hybinette, and iliac crest groups, but not for the Rockwood capsular shift. However, a difference between the operative subgroups was not observed for both analyses.

Giving a prospect regarding development of OA after a shoulder dislocation might be challenging for professionals at the outpatient clinic. Besides from the general risk factors for development of OA, professionals are confronted with the multiple treatment options. Bony augmentation procedures, such as the Latarjet or Iliac crest bone graft procedure, are usually indicated when more glenoid bone loss is present [51]. Excessive glenoid bone loss is often the result of multiple dislocations and this is associated with development of OA [25, 26, 28]. One of the factors that might explain the association between multiple dislocations and development of OA is changes in morphology of the glenoid as a result of multiple dislocations [23]. However, based on the data of this systematic review, these types of surgeries, which are used to treat excessive glenoid bone loss, do not seem to contribute to a higher proportion of OA. The Latarjet procedure even showed a lower proportion and degree of OA compared to the other treatment types. As the development of OA is associated with multiple dislocations, recurrent dislocation after operation may contribute to a higher degree of OA at follow-up. Both the Latarjet procedure and the Iliac crest bone graft procedure have low recurrence rates, showing a weighted recurrence rate of 2% and 3% respectively. The bony augmentation procedures may offer more stability compared to other procedures, which could prevent micro instability and development of OA in the long term [7, 36]. However, the Bankart repair, showing a wide range of 14% to 86% OA, may have a wider indication range compared to the bony augmentation procedures.

The difference in pooled proportion of OA between the Latarjet procedure and the Iliac crest bone graft procedure is remarkable with values of 34% and 61%, respectively. Since both procedures use a bone graft to stabilize the shoulder, this difference could be based on coincidence [21, 42, 61]. The sample sizes of the iliac crest studies are smaller, and two of the ten studies showed proportions of 65% and 71% for the Latarjet procedure, as well. Furthermore, the meta-analyses did not show a difference between proportions OA of preoperative and follow-up data or proportions of OA for the injured compared to the contralateral shoulder for the Latarjet procedure compared to other procedures. However, at follow-up, the degree of OA seems lower following the Latarjet procedure. An explanation might be that the Latarjet studies started with lower degrees of pre-operative OA.

OA can be classified through multiple classification methods of which the Samilson–Prieto method is most commonly used [11, 55, 56]. These methods determine severity, which is generally based on the presence of osteophytes, joint space irregularity, and joint space narrowing. The reliability of the Samilson–Prieto classification, which is the most commonly used method, is generally good [10, 17]. As the Samilson–Prieto classification is based on the presence and size of osteophytes on the inferior glenoid, Ilg et al. question if damage to the inferior part of the glenoid, such as glenoid bone loss, may lead to a higher classification [33]. Both Ilg et al. and Hovelius et al. stated that the Samilson–Prieto classification alone might not be enough to reliably determine OA [27, 33]. Furthermore, it is unclear whether the type of surgical procedure influences the Samilson–Prieto classification. A bone augmentation procedure adds a bone block to the inferior part of the glenoid, which may result in a different classification as well. For example, the Iliac crest bone graft procedure had many patients classified as grade I with the Samilson–Prieto classification. The difference between grade 0 and grade I may illustrate that it can be difficult to assess OA with a bone graft that is attached to the inferior part of the glenoid [2].

There were some limitations to this systematic review. The included studies are primarily of low level of evidence with mediocre scores in the MINORS quality assessment. Most of these studies were performed with a retrospective design. The length of follow-up varies widely, posing a challenge with regard to drawing firm conclusions. However, a strength of this systematic review includes a systematic search and selection process according to the PRISMA guidelines. Furthermore, a large amount of data and sufficient studies made a quantitative meta-analysis feasible.

Many uncertainties remain regarding degree of OA following an anterior shoulder dislocation. The presented data show that the type of operative procedure may have little effect on the development of OA and that a Latarjet procedure may even show a lower degree of OA compared to other treatment options. Future studies should focus on shoulder stability as a factor in developing OA [36]. There appear to be little changes in proportion of OA between 5 and 35 years of follow-up. Studies with short- to mid-term follow-up of 10 years might be valuable in identifying patients that are prone to developing OA in an early stage. Finally, few studies exist that consider surgical options that reduce instability by treating a Hill–Sachs lesion, such as a remplissage in addition to the Bankart repair [1]. The only study that was found in this systematic review that treated a Hill–Sachs lesion was Weber’s rotational osteotomy. These treatment options are underexposed and they require long-term studies to determine the proportion and degree of OA, as well.

## Conclusions

Non-operative and operative treatments show similar OA proportions at any point of follow-up. The hypothesis that shoulders showed a higher proportion or degree of OA following operative treatment compared to non-operative treatment is not supported by the data. Operative treatment according to the Latarjet procedure results in a lower degree of OA compared to other treatments, including non-operative treatment.

## Electronic supplementary material

Below is the link to the electronic supplementary material.

(DOCX 102 kb)

## Abbreviations

OA: Osteoarthritis
PRISMA: Preferred Reporting Items for Systematic Reviews and Meta-Analyses
MINORS: Methodological index for non-randomized studies
