# Supplementary material for: Treatment type may influence degree of post-dislocation shoulder osteoarthritis: a systematic review and meta-analysis
Source: Knee Surg Sports Traumatol Arthrosc. 2020 Sep 16;29(7):2312–24. doi: 10.1007/s00167-020-06263-3 (PMC8225537; doi:10.1007/s00167-020-06263-3)
Supplement: Supplementary file 1 — (DOCX 102 kb) [file 167_2020_6263_MOESM1_ESM.docx]

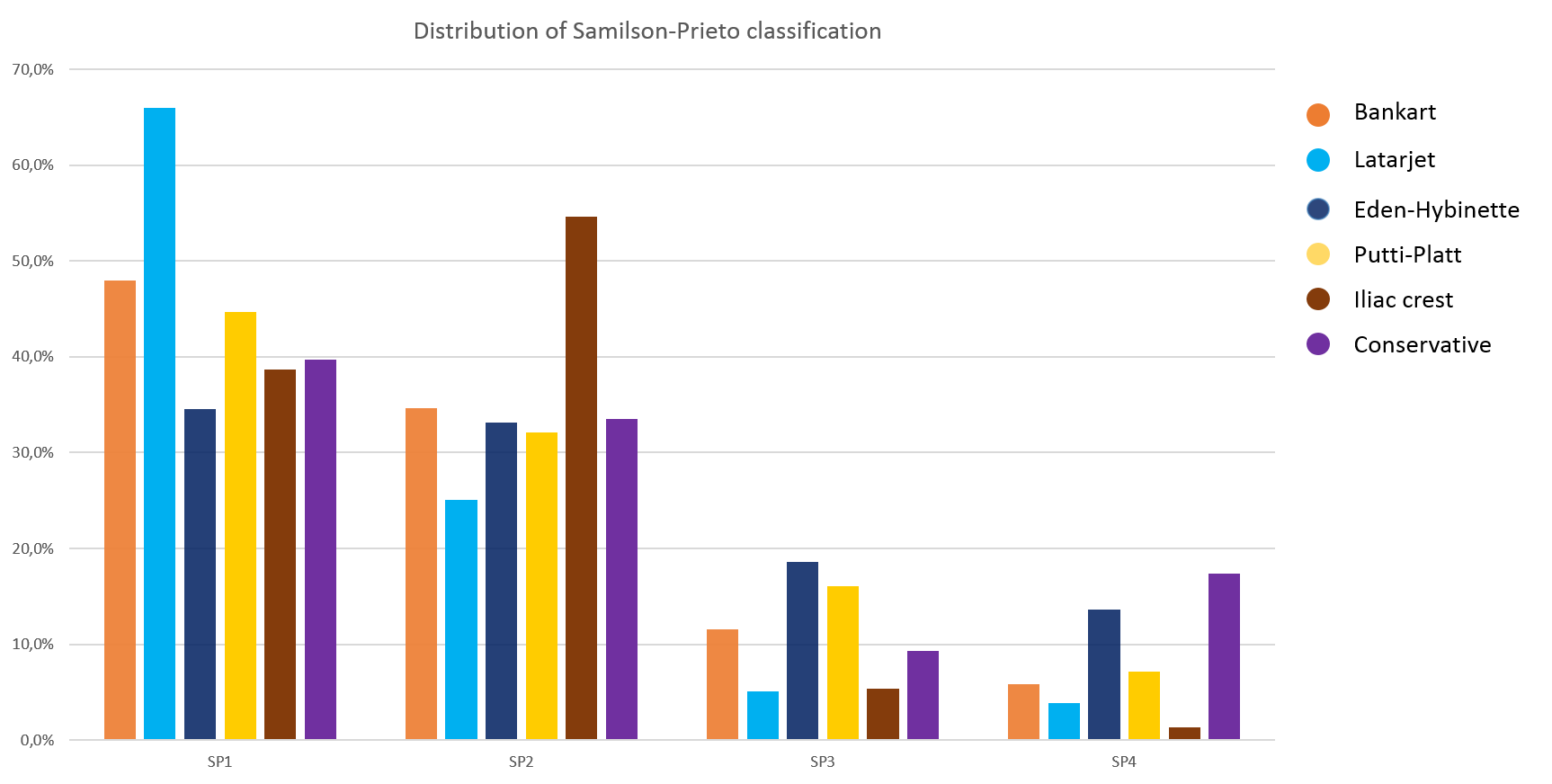
**Appendix 1 Distribution of Samilson-Prieto classification.** This figure shows the distribution of the Samilson-Prieto classifications for treatment options with a pooled sample size of more than 50. SP = Samilson-Prieto

**Appendix 2 Search terms Pubmed/Medline.**

(((((((("Shoulder Dislocation"[Mesh] OR "Bankart Lesions"[Mesh])) OR (("Shoulder"[Mesh] OR "Shoulder Joint"[Mesh]) AND "Joint Instability"[Mesh])) OR ((shoulder*[tiab] OR glenohumeral[tiab]) AND (instab*[tiab] OR dislocat*[tiab] OR luxat*[tiab])))) AND (("Orthopedic Procedures"[Mesh] OR "surgery" [Subheading] OR surg*[tiab] OR repair*[tiab]))) AND ((("Arthritis"[Mesh] OR osteoarthr*[tiab] OR arthrosis[tiab] OR arthritis[tiab] OR long term[tiab] OR follow up[tiab])) OR ((osteo*[tiab] AND arthr*[tiab]) OR arthropath*[tiab])))) AND (english[Language] OR dutch[Language] OR german[Language]) NOT

("Editorial" [Publication Type] OR "Comment" [Publication Type] OR editorial[ti] OR comment[ti])

NOT ("Animals"[Mesh] NOT "Humans"[Mesh])

**Appendix 3 Search terms EMBASE.**

| # | Searches | Results |
| --- | --- | --- |
| 1 | shoulder dislocation/ or Bankart lesion/ | 6492 |
| 2 | ((shoulder* or glenohumeral) and (instab* or dislocat* or luxat*)).ti,ab,kw. | 11764 |
| 3 | 1 or 2 | 13860 |
| 4 | exp orthopedic surgery/ or surgery.fs. or (surg* or repair*).ti,ab,kw. | 4177706 |
| 5 | exp arthritis/ or arthropathy/ | 508788 |
| 6 | (osteoarthr* or arthrosis or arthritis or arthropath* or long term or follow up).ti,ab,kw. | 2536715 |
| 7 | (osteo* and arthr*).ti,ab,kw. | 71534 |
| 8 | 5 or 6 or 7 | 2727100 |
| 9 | 3 and 4 and 8 | 3673 |
| 10 | limit 9 to conference abstract status | 332 |
| 11 | 9 not 10 | 3341 |
| 12 | limit 11 to (dutch or english or german) | 3026 |
| 13 | editorial/ or erratum/ or letter/ or exp conference paper/ or (editorial or letter).ti. | 2696934 |
| 14 | 12 not 13 | 2938 |
| 15 | animal/ not human/ | 1454804 |
| 16 | 14 not 15 | 2927 |

**Appendix 4 Search terms Cochrane.**

#1 (shoulder dislocation OR bankart lesion*):ti,ab,kw 491

#2 ((shoulder* OR glenohumeral) AND (instab* OR dislocat* OR luxat*)):ti,ab,kw 688

#3 #1 or #2 694

#4 (orthopedic procedure* OR surg* OR repair*):ti,ab,kw 229379

#5 ((osteo* AND arthr*)):ti,ab,kw 8609

#6 (osteoarthr* OR arthrosis OR arthritis or arthropath* or long term or follow up):ti,ab,kw 299376

#7 #5 or #6 299831

#8 #3 and #4 and #7 in Cochrane Reviews, Trials 185
